# Efficacy of Dialectical Comprehensive Treatment of Traditional Chinese Medicine in Patients with Chronic Stable Heart Failure: A Randomized Controlled Trial

**DOI:** 10.1155/2022/5408063

**Published:** 2022-05-12

**Authors:** Lixiang Yang, Yuanyuan Chen, Fei Lou, Xiaoxia Zhao, Jia Zhou

**Affiliations:** ^1^Department of Cardiology, Hangzhou Fuyang Hospital of Traditional Chinese Medicine, Hangzhou, China; ^2^Department of Geriatrics, Hangzhou TCM Hospital Affiliated to Zhejiang Chinese Medical University, Hangzhou, China; ^3^Department of Acupuncture and Rehabilitation, Hangzhou Fuyang Hospital of Traditional Chinese Medicine, Hangzhou, China; ^4^Rehabilitation Centre, Zhejiang Hospital Affiliated to Zhejiang University, Hangzhou, China

## Abstract

The treatment of chronic stable heart failure (CSHF) with integrated traditional Chinese and Western medicine has been of wide concern. We mainly discuss the clinical efficacy of TCM decoction combined with acupuncture and moxibustion (A&M) in CSHF treatment on the basis of syndrome differentiation and treatment (SDT). The control group was given conventional cardiac rehabilitation (CCR), and the treatment group was given TCM decoction combined with A&M treatment based on SDT on the basis of conventional cardiac rehabilitation. The clinical efficacy and cardiopulmonary exercise testing (CPET) indicators were evaluated. Left ventricular ejection fraction (LVEF), NT-proBNP, myocardial ischemia threshold (MIT), and 6-minute walking distance (6MWD) were measured by ultrasound, ELISA, electrocardiogram, and 6MWD test. After treatment, the clinical efficacy, LVEF, and 6MWD of the treatment group were better than in the control group. The NT-proBNP plasma level and MIT in the treatment group were lower than in the control group. The treatment group had enhanced AT, VO_2_ Peak, VO_2_ Peak/HR, and Peak power and decreased resting systolic pressure and peak systolic pressure, and the difference was statistically significant. Dialectical comprehensive treatment of TCM could effectively improve cardiac function and clinical treatment effect, which was worthy of clinical application.

## 1. Introduction

Among cardiovascular diseases, heart failure (HF) is a kind of chronic and progressive clinical syndrome in which the cardiac output cannot meet the metabolic needs of the body due to abnormal cardiac structure or function. Clinically, it is characterized by impaired ejection ability, pulmonary congestion, dyspnea, and insufficient perfusion of organs and tissues [1]. Chronic heart failure (CHF) is the most common type of heart failure in the clinic, which is the terminal stage of most cardiovascular diseases. CHF can be caused by various factors (acute events or chronic diseases), such as hypertension, coronary heart disease, diabetes, and viral infections [2–4]. Chronic stable HF (CSHF) is defined when the symptoms of CHF are stable for more than 1 month. CSHF has a high prevalence and morbidity, which seriously affects the work and life of patients and even threatens their life safety [5]. The death modes of CHF can be mainly divided into three types: (1) unexpected sudden death; (2) sudden death on the basis of deterioration of cardiac function; (3) death caused by various complications [6, 7]. Comprehensive analysis of many clinical studies has corroborated that ACE inhibitors, *β*-adrenergic receptor blockers, cardiotonics, vasodilators, and diuretics could ameliorate long-term prognosis of CHF patients, enhance patient comfort, and attenuate the risk of death and hospitalization [8–10]. However, due to the adverse reactions of multiple drugs, drug contraindications, and difficulty in improving symptoms as a whole, its clinical application is limited. In view of the severe situation of CSHF development in China as well as worldwide, it is necessary to explore reasonable and effective treatment methods for the prevention and treatment of CSHF.

The treatment of CHF by traditional Chinese medicine (TCM) can be traced back to 1000 years ago. The original chronicles on prevention and therapy for CHF were discovered in the book conceived by Sun Simiao, a proverbial ancient Chinese doctor [11]. Syndrome differentiation and treatment (SDT) is the basic principle of Chinese medicine treatment. The integration of disease and syndrome is a pivotal principle of Chinese medicine clinical treatment. Syndrome originates from Chinese medicine, which is a reaction state that describes the essential organic relationship of disease location, etiology, disease nature, disease situation, and the strength of the body's disease resistance ability in a certain stage of the disease process, and it can be manifested as clinically observable symptoms. SDT is a comprehensive analysis of the clinical information acquired from the four diagnostic methods of Chinese medicine (looking, listening, asking, and feeling the pulse) and serves as the guidance of prescription principles [12]. Many clinical researches have manifested the efficacy of TCM on HF [13–15]. However, in these studies, the symptoms of HF patients were stable, and the formulation of TCM was in the form of granules, pills, or injection. They cannot fully reflect the efficacy of SDT in Chinese medicine.

At present, the treatment of CHF with integrated traditional Chinese and Western medicine (TCWM) is widely popular in China. Of the 684 randomized controlled trials, 442 studies (64.62%) used integrated TCWM in the treatment group, which aimed to maximize the advantages of the two therapies and weaken the incidence of adverse reactions [16]. Therefore, in this research, on the basis of conventional cardiac rehabilitation (CCR) (Western medicine treatment, health education, and cardiovascular exercise testing (CPET)), TCM decoction, acupoint acupuncture, acupoint moxibustion, and auricular acupuncture were given according to SDT to evaluate the clinical efficacy and the changes of related indexes of the two groups before and after treatment. This study provides objective basis and new ideas for TCM in the treatment of CHF.

## 2. Materials and Methods

### 2.1. Ethics Statement

Our experimental protocol has been empowered by the ethics committee of Hangzhou Fuyang Hospital of Traditional Chinese Medicine (2019060075). This study complies with the principles of the Helsinki Declaration and the principles of good clinical practice. All participants voluntarily signed informed consent.

### 2.2. Clinical Information

A total of 90 CSHF patients admitted to our hospital from October 2019 to October 2020 were included in this study. The grouping method adopts the random table method. Independent statisticians were doctors (*n* = 3) from Hangzhou Fuyang Hospital of Traditional Chinese Medicine who did not know the experimental design and utilized the statistical analysis system (SAS) to generate random tables. Ninety patients were randomly assigned to the cardiac rehabilitation group (control group, *n* = 45) and the Chinese medicine dialectical comprehensive group (treatment group, *n* = 45) at a ratio of 1 : 1. The general clinical data of the two groups of patients are shown in Table 1. There were no statistically significant differences in gender, age, education, New York Heart Association (NYHA) cardiac function classification, and underlying diseases between the two groups.

### 2.3. Inclusion Criteria

The inclusion criteria were as follows [14, 17]: NYHA functional classification of II–IV; participants with qi deficiency and blood stasis type, yang deficiency and blood stasis type, or blockade of phlegm-turbidity type based on SDT; patients with diagnosis for CHF confirmed by X-ray and coronary angiography; left ventricular ejection fraction (LVEF) ≤40% and N-terminal pro-B-type natriuretic peptide (NT-proBNP) ≥450 pg/mL; and the subjects or their family members are informed and voluntarily participate in clinical research.

### 2.4. Exclusion Criteria

The exclusion criteria were stated as follows [14, 18]: (i) people with allergic constitution and allergy to drugs; (ii) patients with death-prone diseases such as cardiogenic shock, infarct cardiomyopathy, constrictive pericarditis, etc.; (iii) poor compliance and noncompliance with treatment requirements and patient's compliance when measured with the Eight-Item Morisky Medication Adherence Scale (MMAS-8) [19] had a score less than 6 points indicating poor compliance; (iv) those who cannot determine the efficacy or have insufficient data due to various reasons; (v) CHF caused by the failure of vital organs such as the lung, kidney, liver, and other important organ functions; (vi) patients with severe deafness and consciousness disorders; (vii) women who are preparing for pregnancy or are pregnant or lactating.

### 2.5. Diagnostic Criteria of Western Medicine

The diagnosis of Western medicine was made according to the Chinese guidelines for the diagnosis and treatment of heart failure in 2018 [20].

### 2.6. Diagnostic Criteria of Chinese Medicine

The diagnosis of Chinese medicine was made based on the expert consensus on diagnosis and treatment of TCM in CHF in 2002 and guiding principles for the clinical study of new drugs in TCM released in 2014 [21, 22].

### 2.7. Therapeutic Methods

Patients in both groups were treated according to the Chinese guidelines for the diagnosis and treatment of heart failure in 2014 [23]. In the control group, patients were subjected to Western medicine treatment, health education, and CPET as below: (1) patients received the low-salt and low-fat diabetic diet and underwent weight loss, with daily monitoring of blood pressure and blood glucose. Besides, patients received furosemide tablets (Shanghai Zhaohui Pharmaceutical Co., Ltd., H31021074), 20 mg qd; spironolactone tablets (Zhejiang Yatai Pharmaceutical Co., Ltd., H33020111), 20 mg qd; isosorbide mononitrate tablets (Lunan Beite Pharmaceutical Co., Ltd, H10940039), 20 mg bid; atorvastatin calcium tablets (Pfizer Pharmaceuticals Limited, H20051408), 20 mg qn; aspirin enteric-coated tablets (Bayer HealthCare Manufacturing S.r.l., H20160684), 100 mg qd; metoprolol succinate sustained-release tablets (AstraZeneca AB, Sweden, H20140780), 0.5 tablet qd; and irbesartan tablets (Hanhui pharmaceuticals co., LTD, H20040996), 150 mg qd, orally. The above-mentioned drugs were administered in a reasonable combination during the course of treatment and continued for 8 weeks. (2) At the same time of medication, health education was conducted for each patient, including introduction of the cardiac rehabilitation department, the rehabilitation process, popular science knowledge and education of coronary heart disease, hypertension, diabetes, and related drugs, safety education, fall prevention education, self-first aid education, fatigue management, diet self-management, etc. Health education was conducted three times a week for 10 minutes each. (3) CPET was performed for each patient. All subjects in this study were tested using the CPET system (Schiller CS-200, Switzerland). The exercise equipment was a power bicycle, an incremental power exercise program was adopted, and a suitable power load was selected according to the subject's cardiopulmonary function status and daily activity ability. Under the supervision of doctors, the maximum number of symptom-limited CPETs was carried out to encourage patients to exercise until the respiratory exchange rate exceeded 1.05. Meanwhile, the gas exchange index, heart rate, blood pressure, electrocardiogram, and oxygen saturation were measured and recorded with each breath. Patients exercised for 40 minutes each time (5 minutes during warm-up, 30 minutes during exercise, and 5 minutes during recovery), 3 times per week.

On the basis of the control group, the treatment group was given TCM decoction orally, acupoint acupuncture, acupoint moxibustion, and ear acupuncture treatments according to SDT. Firstly, patients with qi deficiency and blood stasis were treated with Baoyuan Decoction combined with Taohong Siwu Decoction. TCM prescription is as follows: *Astragali Radix* 18 g, *Paeoniae Rubra Radix* 10 g, *Codonopsis Radix* 15 g, *Ziziphi Spinosae Semen* 15 g, *Angelicae Sinensis Radix* 10 g, *Dioscoreae Rhizoma* 15 g, *Salviae Miltiorrhizae Radix et Rhizoma* 15 g, *Ophiopogonis Radix* 12 g, *Schisandrae chinensis Fructus* 10 g, *Cinnamomi ramulus* 15 g, *Carthami flos* 6 g, *Persicae Semen* 6 g, and *Glycyrrhizae Radix et Rhizoma* 6 g. On the basis of the above-given administration, patients received acupuncture (Xinshu (BL 15), Xuehai (SP 10), Danzhong (RN 17), or Yinxi (HT 06)), moxibustion (Qihai (RN 6) or Guanyuan (RN 4)), and ear acupuncture treatment (heart, small intestine, sympathetic, or subcortical). Secondly, patients with yang deficiency and blood stasis were treated with Zhenwu Decoction combined with Xuefu Zhuyu Decoction. TCM prescription is as follows: *Poria* 15 g, *Achyranthis Bidentatae Radix* 12 g, *Aconiti lateralis praeparata Radix* 6 g, *Zingiberis Rhizoma* 3 g, *Paeoniae Alba Radix* 12 g, *Rehmanniae Radix* 10 g, *Angelicae Sinensis Radix* 10 g, *Carthami flos* 6 g, *Persicae Semen* 6 g, *Aurantii Fructus* 6 g, *Paeoniae Rubra Radix* 6 g, *Platycodonis Radix* 6 g, *Chuanxiong Rhizoma* 9 g, *Rhizoma Atractylodis Macrocephalae* 15 g, and *Bupleuri Radix* 6 g. On the basis of the above-given administration, patients received acupuncture (Neiguan (PC 6), Tongli (HT 5), Xuehai (SP 10), or Xinshu (BL 15)), moxibustion (Shenque (RN 8) or Shenshu (BL 23)), and ear acupuncture treatment (heart, small intestine, sympathetic, or subcortical). Thirdly, patients with a syndrome of blockade of phlegm-turbidity were treated with Gualou Xie Banxia Decoction. TCM prescription is as follows: *Semen Trichosanthis* 12 g, *Pericarpium Trichosanthis* 12 g, *Allii Macrostemonis Bulbus* 10 g, *Rhizoma Pinelliae* 12 g, *Codonopsis Radix* 15 g, *Chuanxiong Rhizoma* 9 g, *Poria* 15 g, *Cinnamomi Ramulus* 6 g, and *Glycyrrhizae Praeparata cum Melle Radix et Rhizoma* 6 g. On the basis of the above-given administration, patients received acupuncture (Taiyuan (LU 9), Fenglong (ST 40), Juque (RN14), or Jianshi (PC5)), moxibustion (Shenque (RN8) or Shuifen (RN 9)), and ear acupuncture treatment. Ear acupuncture treatment was mainly performed with the heart, small intestine, sympathetic and subcortical regions, supplemented by the lung, liver, chest, antihypertensive sulcus, excitement, and acupuncture strong stimulation. In the above acupoint therapy, 3–5 acupoints were selected each time, the needle was retained for one hour, once every other day, and a period of two weeks was a course of treatment.

The above-mentioned Chinese herb plants were obtained from the Chinese Medicine Pharmacy of Zhejiang Hospital and were uniformly decocted by the decocting room, bagged and sealed. One dose per day was taken twice in the morning and evening for continuous treatment for 8 weeks.

## 3. Observation Indicators and Methods

### 3.1. Criteria of Curative Effects

The efficacy standard of cardiac function was evaluated in accordance with the guiding principles for clinical research of new drugs of traditional Chinese medicine. CHF basic control or cardiac function improvement of more than grade 2 was markedly effective; the improvement of cardiac function by grade 1 but less than grade 2 was effective; the improvement of cardiac function of less than 1 grade was invalid. The total effective rate is the percentage of effective and markedly effective people.

### 3.2. Ultrasound and Holter

Before the cardiac rehabilitation program and after 8 weeks of treatment, the patients underwent color Doppler echocardiography to assess cardiac function. Color Doppler ultrasound (EPIQ7) was bought from Philips and utilized to estimate LVEF. The dynamic electrocardiogram system (DigiTrak XT) was bought from Philips and utilized to examine myocardial ischemia threshold (MIT) [21].

### 3.3. Laboratory Examination

Before the cardiac rehabilitation program and after 8 weeks of treatment, fasting venous blood were drawn from the two groups of patients. After treatment, the NT-proBNP ELISA kit (C01XX-1, Meso Scale Discovery, USA) was utilized to determine the level of NT-proBNP *in vivo* based on the manufacturer's instructions [25].

### 3.4. 6-Minute Walking Distance (6MWD)

The 6MWD experiment was conducted in a 30-meter-long corridor. Participants were instructed to walk at a maximum comfortable speed for 6 minutes. In this study, the score was based on the meter of six-minute walk [26].

### 3.5. Evaluation of CPET Indicators

The resting heart rate, peak heart rate, resting systolic pressure, peak systolic pressure, resting diastolic pressure, peak diastolic pressure, anaerobic threshold (AT), peak oxygen uptake (VO_2_ Peak), peak oxygen pulse (VO_2_ Peak/HR), and peak power were measured by CPET in the observation group and the control group.

### 3.6. Analysis of Adverse Reactions

Adverse reactions of the two groups were recorded at any time during treatment.

### 3.7. Statistical Analysis

SPSS 20.0 software was utilized to statistical analysis. The measurement data were expressed as mean ± standard deviation. The *t*-test was employed for comparison between groups. The enumeration data were analyzed with *χ*^*2*^ test. The grade data were analyzed by Ridit, and *P* < 0.05 was considered statistically significant.

## 4. Results

### 4.1. Comparison of Effect on Cardiac Function between the Two Groups

After 8 weeks of treatment in the two groups, the total effective rate of the treatment group was higher than that of the control group and the total effective rate of clinical treatment was 86.67%, which was significantly better than the 66.67% of the control group. The data differences between the groups were highly comparable (*P* < 0.05, Table 2).

### 4.2. Comparison of LVEF and Plasma NT-proBNP between the Two Groups

Before treatment, there was no statistically significant difference in the levels of LVEF and NT-proBNP between the two groups (*P* > 0.05, Table 3). After treatment, the level of plasma NT-proBNP in both groups was lower than that before treatment and the decrease in plasma NT-proBNP in the treatment group was more obvious than that in the control group (*P* < 0.05, Table 3). After treatment, the LVEF of the two groups was higher than that before treatment and the LVEF of the treatment group rose more significantly than that of the control group (*P* < 0.05, Table 3).

### 4.3. Comparison of MIT and 6MWD between the Two Groups

Before treatment, there was no statistically significant difference between the two groups of MIT changes and 6MWD cardiac function indexes (*P* > 0.05, Table 4). After treatment, the change level of MIT in the two groups was lower than before treatment and the change level of MIT in the treatment group decreased more notably than that in the control group (*P* < 0.05, Table 4). After treatment, the 6MWD of the two groups was higher than that before treatment and the 6MWD of the treatment group enhanced more evidently than that of the control group (*P* < 0.05, Table 4).

### 4.4. Comparison of CPET Evaluation between the Two Groups

There was no difference in resting heart rate, peak heart rate, resting diastolic pressure, and peak diastolic pressure before and after treatment between the two groups (Table 5). Before treatment, there was no difference in resting systolic pressure, peak systolic pressure, AT, VO_2_ Peak, VO_2_ Peak/HR, and Peak power between the two groups (Table 5). After treatment, resting systolic pressure and peak systolic pressure of the two groups decreased notably compared with before treatment; AT, VO_2_ Peak, VO_2_ Peak/HR, and Peak power were evidently higher than before treatment (*P* < 0.05, Table 5). More importantly, the downregulation of resting systolic pressure and peak systolic pressure and the upregulation of AT, VO_2_ Peak, VO_2_ Peak/HR, and Peak power in the treatment group were more significant than those in the control group (*P* < 0.05, Table 5).

### 4.5. Comparison of the Incidence and Recurrence Rate of Adverse Reactions between the Two Groups of Patients

There were no obvious adverse reactions in the control group and the treatment group during the trial, and there were no abnormalities in the liver, kidney, or hematopoietic functions.

## 5. Discussion

It was currently considered that coronary heart disease, cardiomyopathy, and myocardial infarction were crucial factors leading to CHF [27–29]. With the development of modern medical technology, the clinical application of TCM is more reasonable and scientific. Many prominent classical prescriptions of TCM have the ascendancy of notable curative effect and fewer adverse reactions and are widely used in CHF [16]. Clinical studies have corroborated that the combination of TCWM has evident curative effect in the treatment of CHF [13, 30]. There is still insufficient testimony to support the efficacy of syndrome differentiation and treatment of Chinese medicine. In TCM treatment, different treatment methods were adopted according to the SDT of patients. This study was the first randomized clinical trial based on CCR to treat CSHF with oral TCM decoction, acupoint acupuncture, acupuncture moxibustion, and ear acupuncture.

TCM is a treatment method based on the theory of yin and yang and the five elements [31]. In TCM, yin and yang are two contradictory but interdependent concepts. In the human body, the “skin,” “back,” and “Qi” belong to the category of yang, and “muscle and bone,” “abdomen,” and “blood” belong to the category of yin [32]. Besides, in TCM, the theory of five elements is used to interpret the relationship between the physiology and pathology of the human body and the natural environment. For example, “Wood” stands for the liver and gall bladder; “Fire” stands for the heart and small intestine; “Earth” stands for the spleen and stomach; “Metal” stands for the lung and large intestine; and “Water” stands for the kidney and urinary bladder [32]. When the human body suffers from a disease, the dynamic balance of yin-yang and five elements are disturbed [33]; therefore, in order to correct this disturbance, TCM adopts an overall method (i.e., SDT) to make a specific treatment plan for the disease [34, 35]. And, the expert consensus on TCM diagnosis and treatment of CHF published in 2014 gradually unified the classification of TCM syndrome types of HF, which divided HF into three basic syndrome types (qi deficiency and blood stasis type, qi and yin deficiency and blood stasis type, and yang qi deficiency and blood stasis type) [36]. In recent years, SDT of TCM has evident advantages and characteristics in the treatment of CHF. According to the pathogenesis of CHF, the clinical manifestations of CHF patients with qi deficiency and blood stasis type are mainly palpitation, short breath, dim and blackish complexion, or chest and flank pain [21]. According to the relevant study, the treatment of Modified Baoyuan Decoction combined with Taohong Siwu Decoction could effectively improve the clinical treatment effect and improve the clinical symptoms of CHF patients with qi deficiency and blood stasis [37]. The treatment of qi deficiency and blood stasis type was to benefit qi for activating blood circulation; thus, we chose Baoyuan Decoction combined with Taohong Siwu Decoction. The clinical manifestations of CHF patients with yang deficiency and blood stasis type are mainly cold chills limbs, palpitations, chest tightness, or chest pain [38]. Zhenwu Decoction is a representative prescription for treating the syndrome of water overflowing due to yang deficiency; Xuefu Zhuyu Decoction is a representative prescription for treating the syndrome of blood stasis in the chest. The treatment of yang qi deficiency and blood stasis type was to benefit qi, warm yang, and activate blood circulation; thus, we chose Zhenwu Decoction combined with Xuefu Zhuyu Decoction. Moreover, the clinical manifestations of CHF patients with blockade of phlegm-turbidity type are mainly headache, sluggish look, limb twitch, and vomiting of phlegm and drool [39]. The study manifested that Gualou Xiebai Banxia Decoction was effective in treating CHF [40]. The treatment of blockade of phlegm-turbidity type was dissipating phlegm for resuscitation and relieving spasm by calming endogenous wind; thus, we chose Gualou Xiebai Banxia Decoction. Patients with CSHF received decoction therapy according to SDT, combined with acupoint acupuncture, acupoint moxibustion, and auricular acupuncture treatment.

Acupuncture and moxibustion (A&M) is a key treatment method based on the theory of Chinese medicine, which acts on certain acupoints on the human body surface through acupuncture and moxibustion and modulate the viscera, qi, and blood, thereby achieving the purpose of preventing and curing diseases [41]. Acupuncture refers to inserting a needle into patients' body at a certain angle under the guidance of TCM theory and using acupuncture techniques such as twisting, lifting, and inserting to stimulate specific parts of the human body [42]. Moxibustion refers to using preheated Chinese mugwort to burn or fumigate on certain acupoints on the body surface, thus producing thermal stimulation to prevent and treat diseases [43]. Among them, Danzhong has the effects of benefiting heart qi and invigorating heart yang; Neiguan and Tongli have the effects of dredging collaterals, which can prevent too much tonic benefit [44]. Many animal experiments have researched the mechanism of A&M in the treatment of HF and discovered that A&M could repress sympathetic nerve activity, prevent fibrosis, modulate inflammation, reduce myocardial damage, restrain myocardial hypertrophy, and reverse ventricular remodeling [45–49]. Scientific research clarified that A&M could ameliorate the heart function of HF patients [45, 50]. In this research, for the first time, we discovered that the overall clinical effective rate of TCM decoction combined with A&M treatment was higher than that of the CCR treatment.

In the process of diagnosis and treatment of CHF patients, the clinical treatment effect of the patients was generally evaluated by LVEF, NT-proBNP, and 6MWT [51]. LVEF in echocardiogram reflects left ventricular function and can guide clinical treatment, which is an objective index to evaluate the therapeutic effect [52]. It is pointed out in the 2017 US HF management guidelines that NT-proBNP can not only be used to diagnose and exclude HF but also it has a very high negative expectation value and can be used for prognostic judgment and risk stratification [53]. One study confirmed that the addition of Shenfu injection on the basis of conventional western medicine treatment is beneficial to ameliorate the heart function of patients with HF, enhance the total clinical effective rate, and improve the heart rate, NT-proBNP level, and 6MWD of patients [54]. Our study demonstrated that TCM decoction combined with A&M treatment evidently elevated the LVEF, NT-proBNP level, and 6MWD of CSHF patients compared with the CCR treatment, revealing that the addition of TCM decoction and A&M together with CCR can further improve the exercise endurance and quality of life of patients with CSHF.

In CCR treatment, we performed CPET and evaluated the cardiopulmonary function status of the control group and the treatment group. Among them, VO_2_ Peak and AT are some of the best indicators to detect early cardiac dysfunction and to evaluate the degree of improvement of cardiac function in HF patients. VO_2_ Peak/HR is the amount of oxygen that can be transported by the heart per stroke, which can reflect the ability of the heart to deliver oxygen per stroke [55–57]. In this research, TCM decoction combined with A&M treatment further improved AT, VO_2_ Peak, VO_2_ Peak/HR, and Peak power and weakened resting systolic pressure and peak systolic pressure in the treatment group than in the control group, indicating that TCM decoction combined with A&M treatment can further improve cardiac function on the basis of CCR.

## 6. Conclusion

Because all the data in this study are from Zhejiang hospitals, there is inevitably selection bias and the results may be affected by the regional climate characteristics. Another limitation of this study is the small sample size, we will expand the sample size and continue clinical observation to obtain more clinical data and research data in the future. In short, on the basis of CCR, TCM decoction combined with A&M treatment according to SDT can effectively ameliorate the heart function of patients and strengthen the clinical treatment effect, which is worthy of clinical application.

## Figures and Tables

**Table 1:** Comparison of general data between the two groups.

Variable	Control group (*n* = 45)	Treatment group (*n* = 45)	*χ* ^*2*^ */t/Z*	*P*
Gender			0.182	0.670
Male	25	27		
Female	20	18		
Age (years)	65.13 ± 3.41	64.24 ± 3.78	1.172	0.244
Course (years)	3.13 ± 1.12	3.25 ± 0.78	−0.559	0.578
Education			−0.601	0.548
Primary school and below	8	10		
Junior high school	13	14		
High school	13	11		
College degree and above	11	10		
NYHA			−0.819	0.413
II	15	13		
III	20	18		
VI	10	14		
Underlying diseases			0.832	0.842
Coronary atherosclerosis	13	15		
Hypertensive heart disease	18	16		
Valvular heart disease	8	6		
Dilated cardiomyopathy	6	8		

**Table 2:** Comparison of effect on cardiac function between two groups (n (%)).

Group	*n*	Excellent	Effective	Invalid	Total effective rate
Control group	45	14 (31.11)	16 (35.56)	15 (33.33)	30 (66.67)
Treatment group	45	24 (53.33)	15 (33.33)	6 (13.33)	39 (86.67)
*χ2*					5.031
*P*					0.025

**Table 3:** Comparison of LVEF and plasma NT-proBNP between the two groups.

Group	*n*	LVEF (%)		NT-proBNP (ng/mL)	
		Before treatment	After treatment	Before treatment	After treatment
Control group	45	40.08 ± 5.29	44.11 ± 6.67^*∗*^	637.38 ± 48.17	406.53 ± 35.67^*∗*^
Treatment group	45	39.21 ± 4.98	50.16 ± 5.57^*∗*^	624.32 ± 50.89	321.77 ± 39.07^*∗*^
*t*		0.804	−4.671	1.251	10.745
*P*		0.424	0.000	0.214	0.000

Compared with before treatment, ^*∗*^*P* < 0.05.

**Table 4:** Comparison of MIT and 6MWD between the two groups.

Group	*n*	MIT (mv)		6MWD (m)	
		Before treatment	After treatment	Before treatment	After treatment
Control group	45	0.22 ± 0.11	0.16 ± 0.09^*∗*^	337.38 ± 48.17	380.53 ± 35.67^*∗*^
Treatment group	45	0.19 ± 0.13	0.11 ± 0.03^*∗*^	324.32 ± 50.89	421.77 ± 39.07^*∗*^
*t*		1.149	3.679	1.249	−5.229
*P*		0.254	0.001	0.215	0.000

Compared with before treatment, ^*∗*^*P* < 0.05.

**Table 5:** Comparison of cardiopulmonary exercise test evaluation between the two groups.

Group	*n*	Resting heart rate (times/min)		Peak heart rate (times/min)		Resting systolic pressure (mmHg)		Peak systolic pressure (mmHg)		Resting diastolic pressure (mmHg)		Peak diastolic pressure (mmHg)		AT (mL/(kg·min))		VO_2_ Peak (mL/(kg·min))		VO_2_ Peak/HR (mL/Time)		Peak power (w)	
		Before treatment	After treatment	Before treatment	After treatment	Before treatment	After treatment	Before treatment	After treatment	Before treatment	After treatment	Before treatment	After treatment	Before treatment	After treatment	Before treatment	After treatment	Before treatment	After treatment	Before treatment	After treatment
Control group	45	75.52 ± 10.74	77.63 ± 10.95	104.46 ± 14.21	109.25 ± 12.33	120.46 ± 16.96	109.93 ± 14.08^*∗*^	155.12 ± 15.31	143.86 ± 19.61^*∗*^	61.67 ± 6.68	63.47 ± 7.02	75.32 ± 7.96	76.33 ± 9.48	10.52 ± 2.12	13.84 ± 2.64^*∗*^	14.86 ± 3.84	18.16 ± 2.04^*∗*^	7.95 ± 1.45	9.52 ± 1.48^*∗*^	88.74 ± 15.36	98.51 ± 10.55^*∗*^
Treatment group	45	75.32 ± 8.48	74.82 ± 11.45	105.92 ± 12.03	105.90 ± 16.42	118.50 ± 113.06	102.83 ± 8.84^*∗*^	156.35 ± 15.98	132.27 ± 14.76^*∗*^	62.36 ± 6.99	65.04 ± 8.06	75.89 ± 6.57	76.75 ± 7.87	10.76 ± 2.20	15.23 ± 2.26^*∗*^	15.12 ± 3.76	19.45 ± 1.85^*∗*^	8.10 ± 1.65	10.85 ± 1.60^*∗*^	85.62 ± 17.20	105.45 ± 8.62^*∗*^
*t*		0.098	1.192	−0.527	1.096	0.614	2.864	−0.373	3.166	−0.472	−0.986	−0.370	−0.233	−0.527	−2.683	−0.325	−3.142	−0.458	−4.093	0.908	−3.417
*P*		0.922	0.236	0.600	0.276	0.541	0.005	0.710	0.002	0.638	0.372	0.712	0.816	0.600	0.009	0.746	0.002	0.648	0.000	0.367	0.001

Compared with before treatment, ^*∗*^*P* < 0.05.

## Data Availability

The analyzed data sets generated during the study are available from the corresponding author on reasonable request.
